# Identification of key immune-related genes associated with LPS/D-GalN-induced acute liver failure in mice based on transcriptome sequencing

**DOI:** 10.7717/peerj.15241

**Published:** 2023-05-05

**Authors:** Ling Chen, Li Yuan, Jingle Yang, Yizhi Pan, Hong Wang

**Affiliations:** 1 Department of Infectious Disease, Zhejiang Hospital, Hangzhou, China; 2 State Key Laboratory for Diagnosis and Treatment of Infectious Diseases, National Clinical Research Center for Infectious Diseases, Collaborative Innovation Center for Diagnosis and Treatment of Infectious Diseases, The First Affiliated Hospital, College of Medicine, Zhejiang University, Hangzhou, China

**Keywords:** Immunity, Acute liver failure, Mice, Transcriptome sequencing, Bioinformatics, Genes

## Abstract

**Background:**

The aim of this study was to identify key immune-related genes in acute liver failure (ALF) by constructing an ALF mouse model for transcriptome sequencing.

**Methods:**

The C57BL/6 mouse with ALF model was induced by lipopolysaccharide (LPS)/ D-galactosamine (D-GalN). After successful modelling, the liver tissues of all mice were obtained for transcriptome sequencing. The key immune-related genes in mice with ALF were identified by differential expression analysis, immune infiltration analysis, weighted gene co-expression network analysis (WGCNA), enrichment analysis, and protein-protein interaction (PPI) analysis.

**Results:**

An LPS/D-GalN-induced ALF mouse model was successfully constructed, and transcriptome sequencing was performed. Significant differences in the proportions of monocytes, macrophages M0, macrophages M1 and neutrophils were shown by immune infiltration analysis, and 5255 genes highly associated with these four immune cells were identified by WGCNA. These immune genes were found to be significantly enriched in the TNF signalling pathway by enrichment analysis. Finally, PPI analysis was performed on genes enriched in this pathway and three key genes (CXCL1, CXCL10 and IL1B) were screened out and revealed to be significantly upregulated in ALF.

**Conclusions:**

Key immune-related genes in ALF were identified in this study, which may provide not only potential therapeutic targets for treating ALF and improving its prognosis, but also a reliable scientific basis for the immunotherapy of the disease.

## Introduction

Acute liver failure (ALF) is a serious clinical syndrome characterized by massive hepatocellular necrosis and severe hepatic impairment in a short period of time in the absence of underlying liver disease (Vento & Cainelli, 2020). The main clinical manifestations of ALF are rapid onset of jaundice, elevated liver function tests, coagulopathy and encephalopathy with acetaminophen overdose and viral infection being the most common causes (Kappus, 2020). As one of the critical diseases with high morbidity and mortality, ALF progresses rapidly, and is often accompanied by hypotension, cardiovascular dysfunction, hepatic encephalopathy, renal insufficiency, *etc*. in its later stages (Montrief, Koyfman & Long, 2019). Liver transplantation is currently the most advanced therapy for ALF, but the therapy has several limitations, including high medical costs, shortage of liver organs, surgical complications, and immunological rejection, and thus liver transplantation therapy is not widely used (Rodgers & Horrow, 2021). Therefore, in order to further improve the treatment and prognosis of ALF, the pathogenesis and therapeutic targets of ALF have been investigated by more and more studies.

Bioinformatics analysis of biomarkers and key genes for relevant diseases has now become a popular area of research today (Schuler et al., 2022). Merrell et al. (2021) analyzed hepatocytes undergoing extensive chromatin and transcriptional changes during biliary reprogramming by RNA-sequencing. In addition, whole genome sequencing has also identified pyruvate dehydrogenase complexes and lactate dehydrogenase as therapeutic targets for ALF (Ferriero et al., 2018). This study was designed to identify key genes in ALF mice by transcriptome sequencing, aiming at providing effective therapeutic targets for the treatment of ALF and improving its prognosis.

Immunity, as an important player in all major systems of the body, essentially refers to the body’s defensive response against xenogenous or mutated autologous components (Udit, Blake & Chiu, 2022). Numerous studies have confirmed that immunity mediates the apoptosis and necrosis of a large number of cells in ALF, and that innate immune cells are a major pathophysiological determinant of liver injury (Agrawal et al., 2021; Zhang et al., 2019). For example, mesenchymal stromal cells (MSCs) can relieve liver injury by inhibiting immune cell activation, thereby improving the prognosis of ALF (Hu, Wu & Li, 2020); and the formation of neutrophil extracellular traps (NETs) has been shown to be significantly associated with the poor prognosis in patients with ALF (Von Meijenfeldt et al., 2022). It is undeniable that targeting ALF from an immune perspective holds great promise. Therefore, this study intended to identify key genes in ALF mice from an immune perspective, so as to provide a reliable scientific basis for the immunotherapy of ALF.

In recent years, as the research on ALF continues to progress, some studies have started to explore the pathogenesis of ALF by establishing ALF mouse models (Fujimoto, Kuwamura & Azuma, 2020), among which the LPS/D-GalN-induced ALF mouse model has been recognized as one of the effective methods for constructing *in vitro* models of ALF (Hao et al., 2020). It is generally established by intraperitoneal injection of LPS+D-GalN in the experimental group of mice, and the successful modelling is judged by liver enzyme activity measurement and HE staining (Gu et al., 2020). Therefore, in this research, we constructed an LPS/D-GalN-induced ALF model in the experimental group of mice, and determined the modelling status using liver enzyme assay and HE staining. After the successful modelling, the liver tissues from all the mice were collected for transcriptome sequencing. We then identified key genes associated with immunity in ALF in mice by differential expression analysis, immune infiltration analysis, weighted gene co-expression network analysis (WGCNA), enrichment analysis and protein-protein interaction (PPI) analysis.

## Materials & Methods

### Modelling of ALF mice

Normal healthy C57BL/6 male mice weighing 21.5-25 g at 5-6 weeks of age were purchased from the Zhejiang Provincial Laboratory Animal Centre and housed at the Hangzhou Medical College Laboratory Animal Centre. The mice were acclimatised for one week prior to the experiment and all were allowed free access to food and water throughout the period of domestication and experimentation. Mice were housed in cages at 22  ± 2 °C with a 12 h light/dark cycle. We randomly divided the 14 mice into two groups, a control group with six mice and an experimental group with eight mice. To construct the model, the mice in the experimental group were injected intraperitoneally with LPS (10 µg/kg) and D-GalN (300 mg/kg), while mice in the control group were injected with normal saline. The mice were euthanized 24 h after such injection by cervical dislocation, and their serum and liver tissues were collected for subsequent testing. The animal experiment was approved by the Animal Welfare Ethics Committee of Zhejiang Experimental Animal Center (IACUC, ZJCLA), the ethical number is ZJCLA-IACUC-20010066. The study followed the Declaration of Helsinki.

### Determination of liver enzyme activity and hematoxylin-eosin staining (H & E staining)

The activity of AST and ALT in the serum was measured using AST assay kit (C010-2) and ALT assay kit (C009-2), respectively. H&E staining of liver tissues was performed according to the instructions of the modified HE staining kit (YT8681; Yita Biological Co., Ltd., Yita, China).

### RNA extraction

After the mice were euthanized, their liver tissues were removed and the RNA in the tissues was extracted for sequencing by the experimental Trizol method.

### RNA sequencing

First, Oligo (dT) magnetic beads were used to separate the mature mRNA from total RNA. Then, the fragmented mRNA was set as template for the synthesis of the first and second strands of cDNA. After being purified, the cDNA was subjected to PCR amplification, and the cDNA library was obtained accordingly. Finally, an Illumina Novaseq 6000 was used for RNA sequencing after quality inspection of the obtained library. The statistical power of this experimental design, calculated inRNASeqPower is 0.88, based on an average sequencing depth of 117X, CV of 0.4.

### Data analysis

FASTP (default parameters) was used for quality control on the original FASTQ data. HISAT2 was applied to compare the data after quality control to reference genome (GRCm39). VM28 GTF file was obtained from GENCODE, and raw Read Counts were obtained by tSEQ-count.

### DEG analysis and enrichment analysis

DEG analysis results were presented by a volcano plot using DESeq2 (|logFC| > 1, *padj* < 0.05). DEG analysis was performed using gencode.vM28.annotation.gtf for annotation and using all annotated genes including ncRNA. ClusterProfiler was used for Gene Set Enrichment Analysis (GSEA) of all the genes. All the genes were subjected to GSEA enrichment analysis based on the logFC results of DESeq2 differential analysis arranged in descending order in the direction ALF-high to ALF-low. GO and KEGG enrichment analysis were performed using ClusterProfiler, *p* < 0.05, with display entries set to 10. The results of GSEA were shown for five pathways, KEGG for 10 or 20, and GO-BP, GO-CC and GO-MF for 10 each.

### Immunoinfiltration analysis

The proportion of immune cells in the samples was analyzed using CIBERSORTX and a *t*-test was performed.

### WGCNA

The Pearson’s correlation coefficient was used to cluster the samples. The soft threshold was set to 8 (R 2 = 0.8) to construct a scale-free network. And the adjacency matrix and the topological overlap matrix were constructed. Then, 15 non-grey modules were identified based on average hierarchical clustering and dynamic tree clipping. Finally, the turquoise and red modules were selected for subsequent analysis based on correlations greater than 0.7 between the modules and ALF, monocytes, macrophages M0, macrophages M1, and neutrophils.

### Enrichment analysis and PPI analysis

The results of WGCNA were intersected with DEGs, subsequently subjected to GO and KEGG enrichment analysis, and the pathway with the smallest p.adjust in the KEGG enrichment analysis was selected for subsequent study. Genes enriched in this pathway were subjected to PPI analysis, and then the results of five algorithms (BottleNeck, Degree, EPC, MCC and MNC) were intersected using the cytohHubba plugin of the Cytoscape software to finally obtain the key genes associated with ALF immunity.

### Statistical analysis

GraphPad 9.0 was used for statistical analysis. Student’s *t*-test was used for difference comparison between the two groups, and *P* < 0.05 suggested a statistically significant difference. Data were expressed as mean  ± standard deviation (SD).

## Results

### Modeling of ALF mice

**Figure 1:**
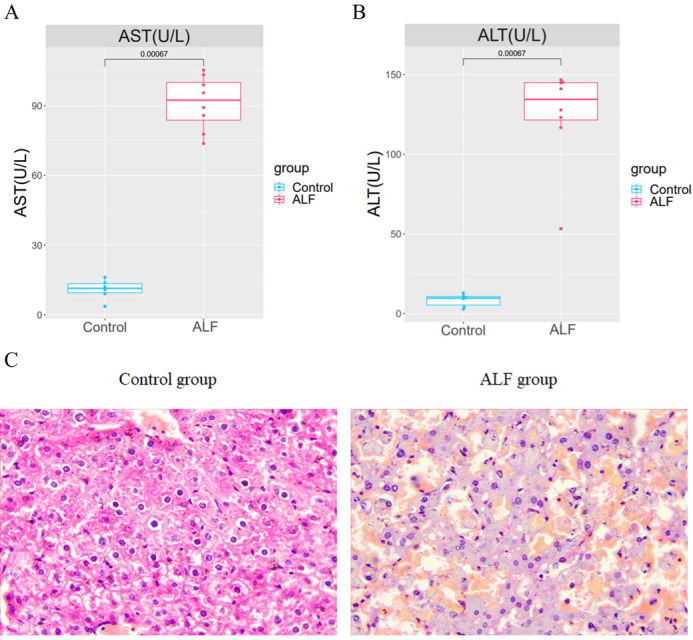
Establishment of the ALF mouse model. (A) Differences of AST and ALT in the serum between ALF group and control group. (B) H&E staining in liver tissues of ALF group and control group (200×).

An LPS/D-GalN-induced ALF model was established by intraperitoneal injection of LPS+D-GalN into eight mice in the experimental group and normal saline injection into 6 mice in the control group. After 24 h of injection, the serum and liver tissues of mice were collected for analysis. The AST and ALT levels in the serum of ALF mice were shown to be significantly higher than those in the serum of the controls (Figs. 1A–1B). The successful modelling establishment was indicated when focal necrosis and structural disorder in the hepatocytes of ALF mice were revealed by H&E staining, accompanied by hepatocellular oedema (Fig. 1C). Then, transcriptome sequencing was performed on the RNA extracted from the liver tissues.

### Identification and functional enrichment analysis of DEGs

**Figure 2:**
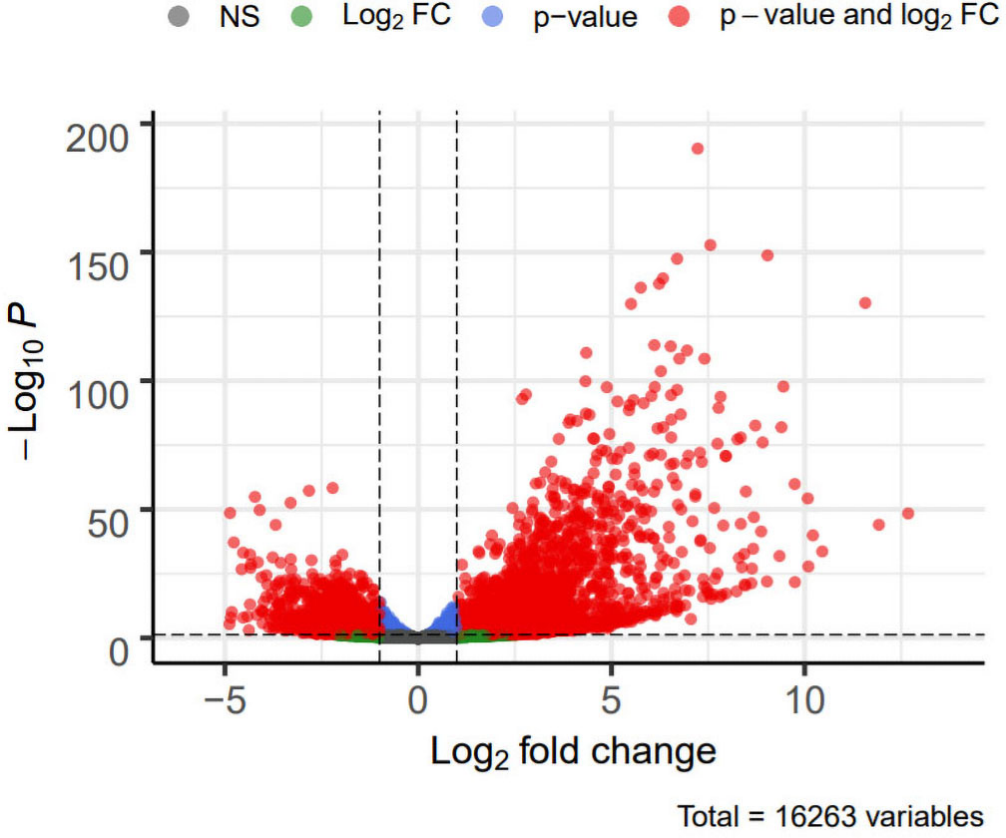
Volcano plot showing DEGs between the ALF group and control group.

**Figure 3:**
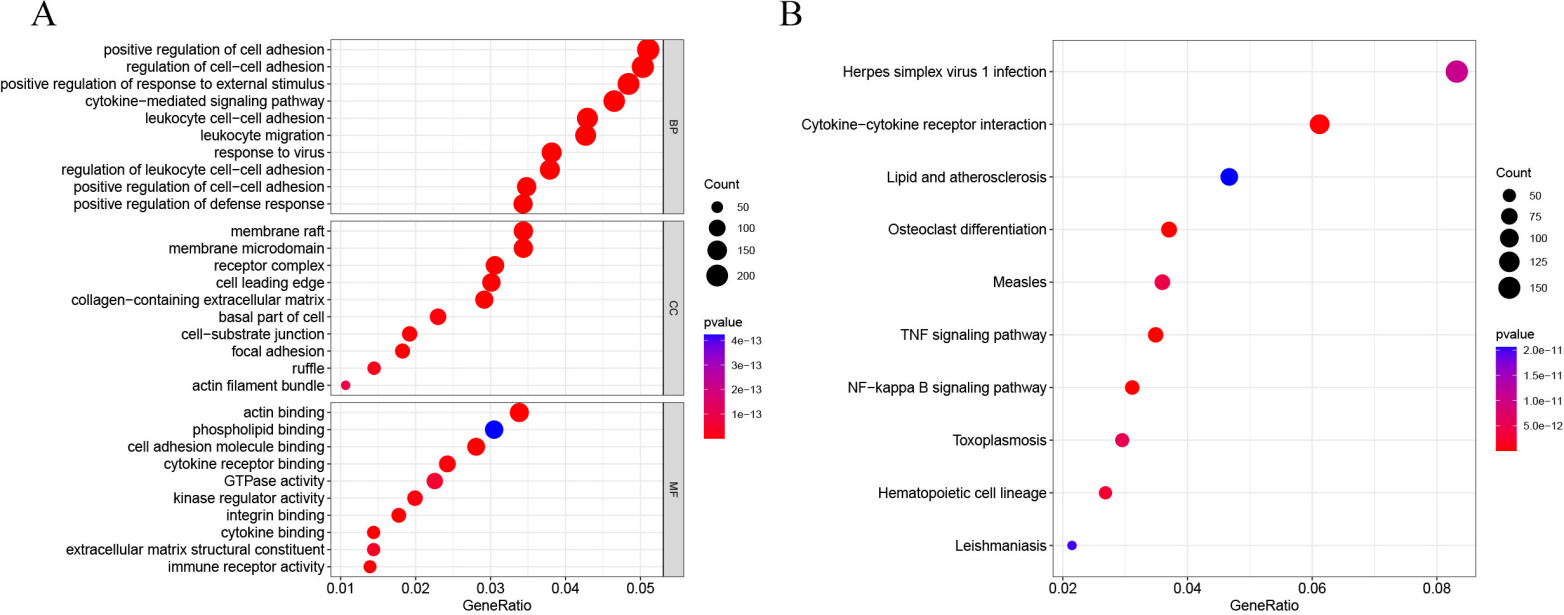
GO and KEGG enrichment analysis. (A) GO enrichment analysis. (B) KEGG enrichment analysis.

DEG analysis results were presented by a volcano plot using DESeq2 (|logFC| > 1, *padj* < 0.05). We identified 4,643 DEGs in the ALF group *versus* the control group, of which 2828 were up-regulated and 1815 were down-regulated (Fig. 2). Genes with adjusted *p* < 0.05 and |logFC| > 1 were marked in red, genes with adjusted *p* < 0.05 and |logFC| ≤ 1 were marked in blue, genes with |logFC| > 1 and adjusted *p* > 0.05 were marked in green, genes with adjusted *p* > 0.05 and |logFC| ≤ 1 were marked in gray. GO and KEGG enrichment analysis showed that the DEGs were enriched in cytokine-mediated signaling pathway, positive regulation of defense response and other immune-related pathways (Figs. 3A–3B).

### Enrichment analysis

**Figure 4:**
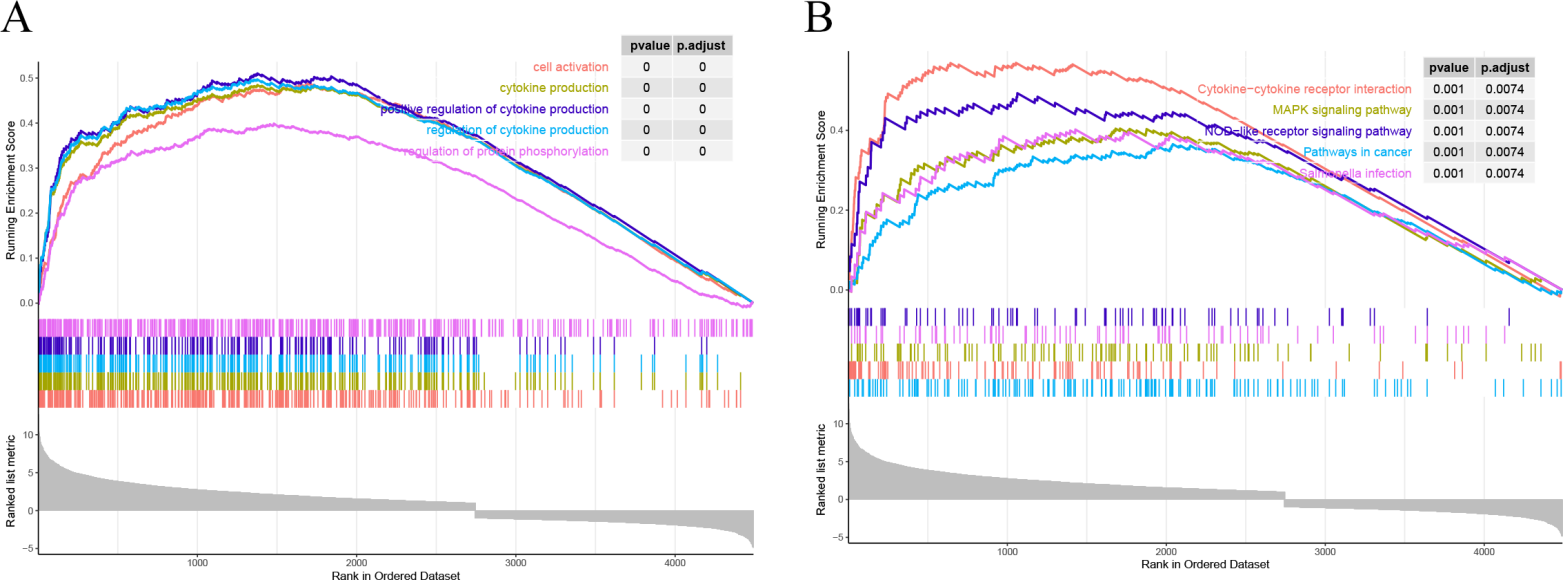
Results of GSEA. (A) GSEA enrichment analysis based on GO. (B) GSEA enrichment analysis based on KEGG.

All the genes were subjected to GSEA based on the logFC results of DESeq2 differential analysis arranged in descending order in the direction ALF-high to ALF-low. GSEA showed that genes in ALF mice were mainly enriched in cytokine-cytokine receptor interaction, cell activation, cytokine production and other pathways (Figs. 4A–4B).

### Proportion analysis of immune cells

**Figure 5:**
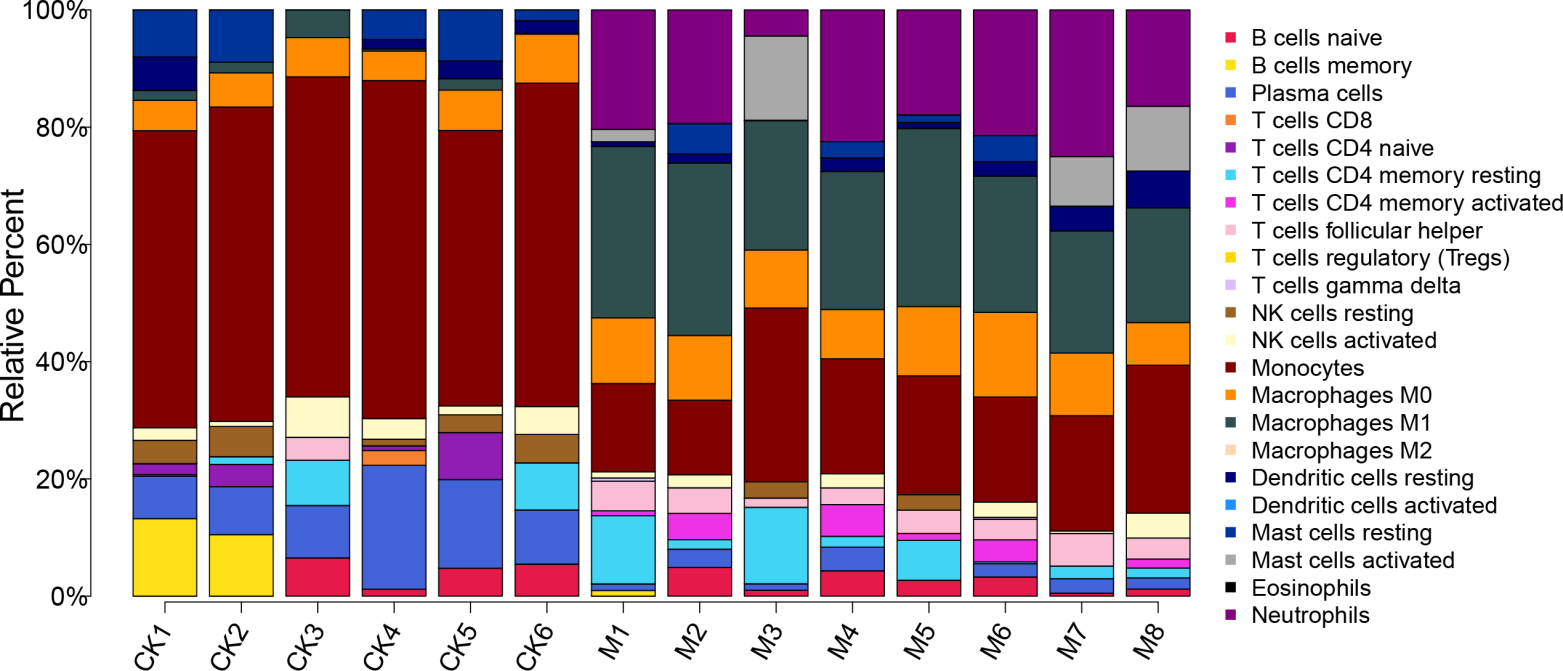
Proportion of 22 immune cell types in the ALF group *vs.* control group.

**Figure 6:**
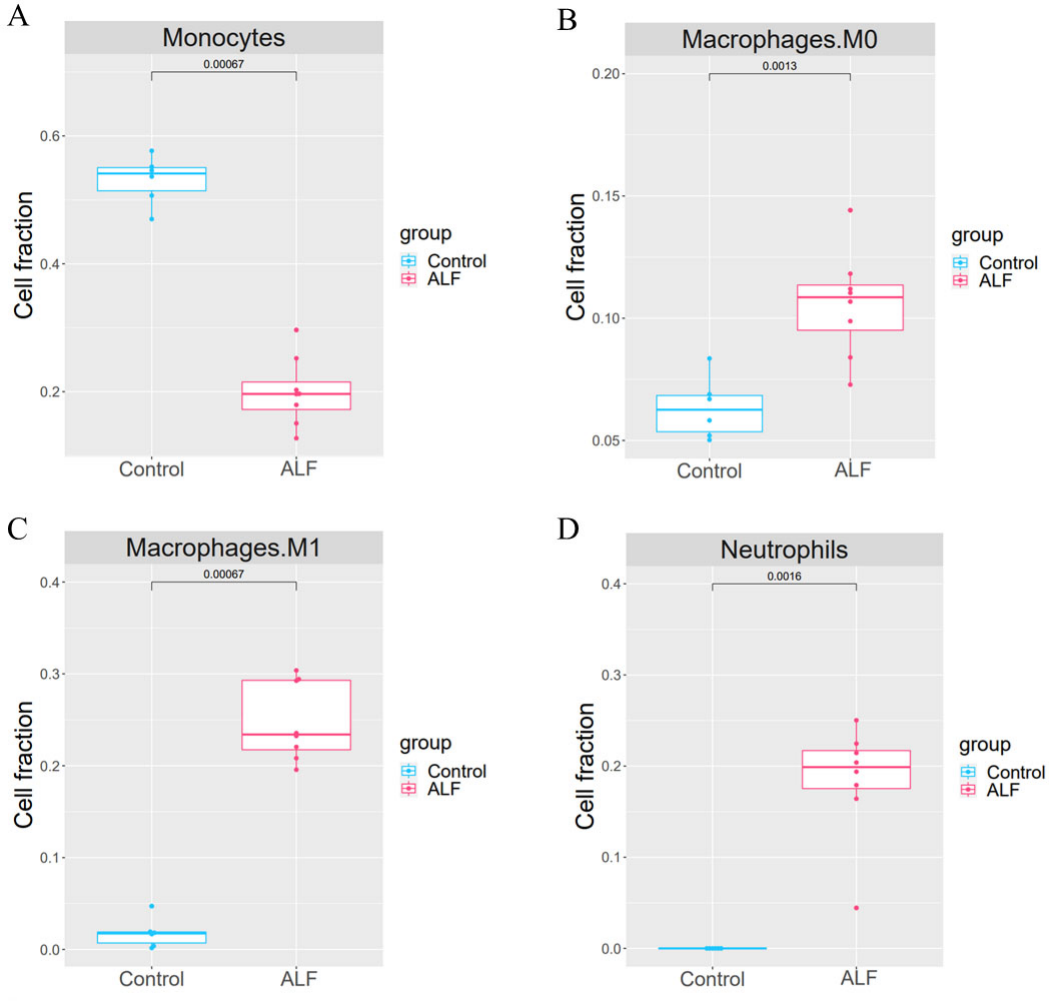
Cell proportion analysis. (A) *t*-test analysis of monocytes. (B) *t*-test analysis of macrophage M0. (C) *t*-test analysis of macrophage M1. (D) *t*-test analysis of neutrophils.

The proportion of 22 immune cells in each sample was calculated using CIBERSORT (Fig. 5). The proportion of Monocytes was significantly lower in ALF mice than that in the control mice, while the proportions of Macrophages M0, Macrophages M1, and Neutrophils in ALF mice were significantly higher than those in the control mice (Figs. 6A–6D).

### WGCNA clustering results

**Figure 7:**
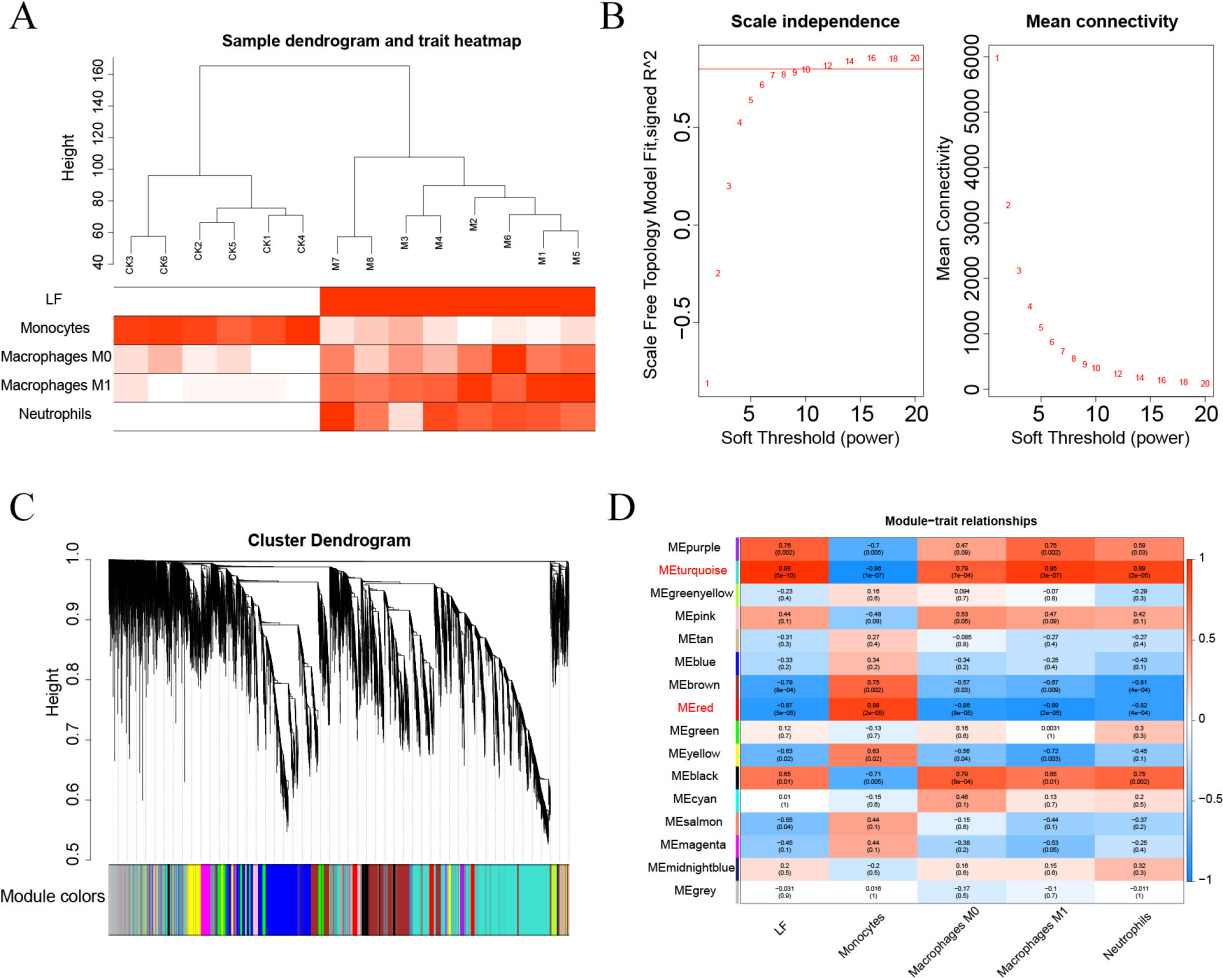
(A–D) WGCNA analysis.

First, the samples were subjected to WGCNA, and then a soft threshold of 16 was selected for weighted co-expression network construction and correlation analysis between modules and features (Figs. 7A–7B). The turquoise and red modules had correlations greater than 0.7 with ALF, Monocytes, Macrophages M0, Macrophages M1 and Neutrophils, which were the highest correlations of all modules (Figs. 7C–7D). Therefore, we selected these two modules for subsequent analysis.

### Enrichment analysis and PPI analysis

**Figure 8:**
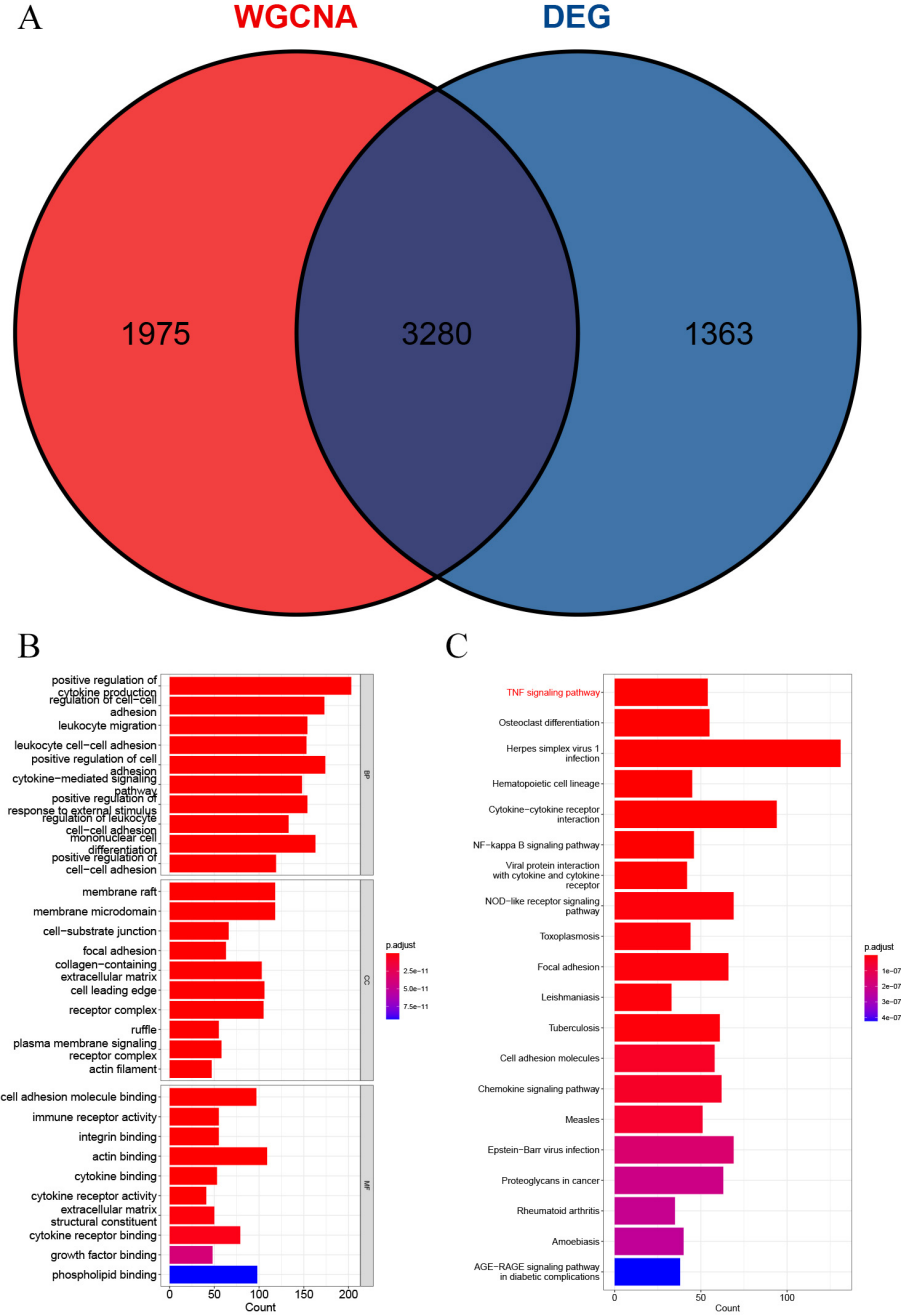
Acquisition of intersecting genes and enrichment analysis. (A) Intersection of WGCNA signature module genes with DEGs. (B) GO enrichment analysis. (C) KEGG enrichment analysis.

**Figure 9:**
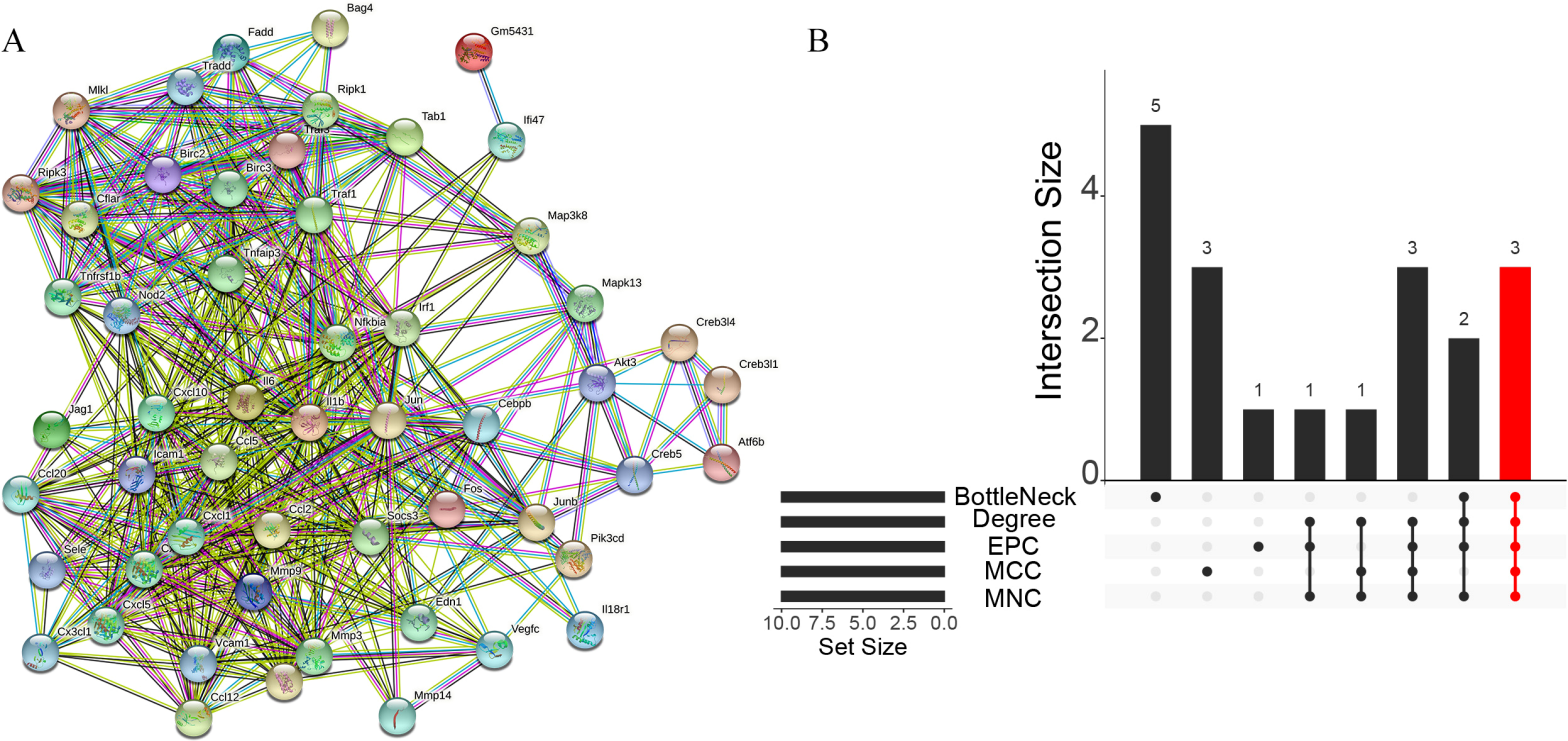
Acquisition of key genes. (A) PPI analysis of genes enriched in the TNF signalling pathway. (B) Upset plot showing the intersection of the results of the five algorithms.

A total of 3,280 immune-related differential genes were obtained by making intersection of the genes with DEGs from the above two modules of WGCNA (Fig. 8A). Then, GO and KEGG enrichment analysis were performed on the intersected genes, and the pathway with the smallest p.adjust in the KEGG enrichment analysis (TNF signaling pathway) was selected for subsequent study (Figs. 8B–8C). Genes enriched in this pathway were subjected to PPI analysis, and then the cytohHubba plugin of Cytoscape software was used to take the intersection of the results of five algorithms (BottleNeck, Degree, EPC, MCC and MNC). Finally, a total of three key genes (CXCL1, CXCL10 and IL1B) were obtained (Figs. 9A–9B). The results of the above five algorithms were detailed in Table S1.

### Expression levels of key genes

**Figure 10:**
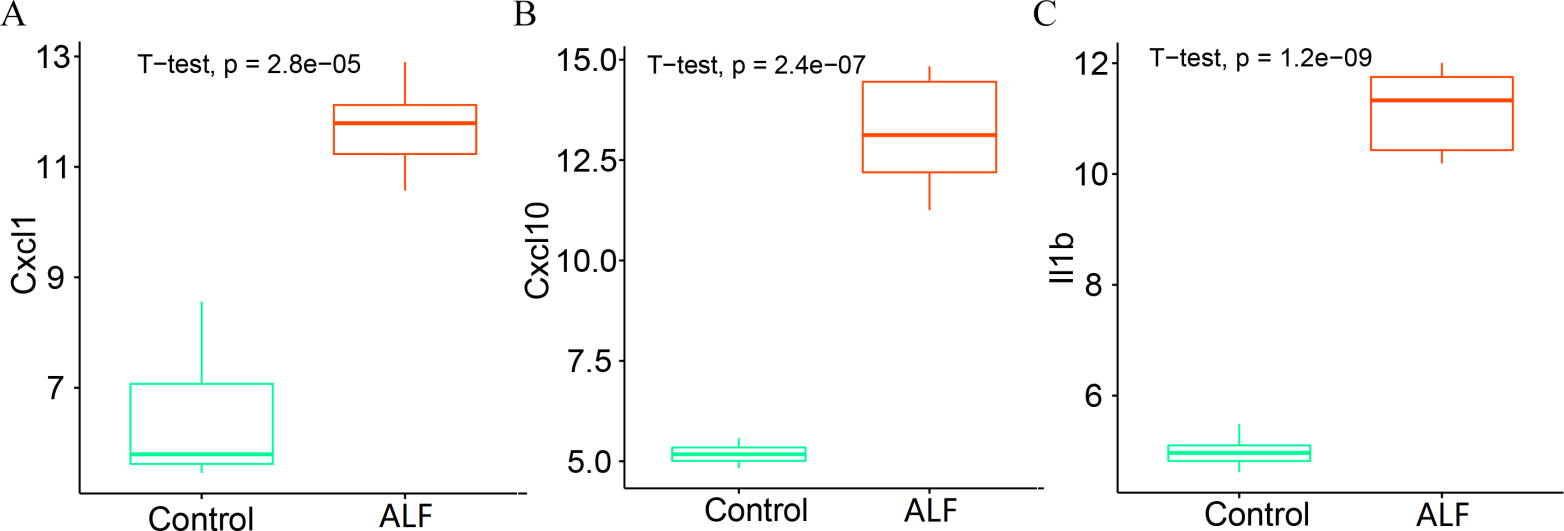
Determination of gene expression level. (A) Expression level of CXCL1. (B) Expression level of CXCL10. (C) Expression level of IL1B.

We further analyzed the expression levels of the three key genes (CXCL1, CXCL10 and IL1B) between the two groups. The box plots showed that the expression levels of all the three key genes were significantly higher in the ALF group than those in the control group (Figs. 10A–10C).

## Discussion

ALF is a group of rare multi-etiological syndromes characterized by loss of synthetic function in the manifestations of jaundice, coagulopathy and hepatic encephalopathy (Dong, Nanchal & Karvellas, 2020). Multi-organ failure often occurs in the final stage of ALF, which is the cause of death (Rajaram & Subramanian, 2018). With the development of relevant supportive treatments and liver transplantation techniques, the survival rate of ALF patients has improved significantly compared to previous periods, but poor prognosis still exists due to the limitations of the ALF treatments (Arshad, Murphy & Bangash, 2020; García-Cortés, Ortega-Alonso & Andrade, 2022). Therefore, more and more studies have been conducted to explore and clarify the pathogenesis of ALF and its therapeutic targets by constructing animal models *in vitro* (Kolodziejczyk et al., 2020). Similarly, in this research, an LPS/D-GalN-induced ALF model was constructed using eight experimental mice, and six healthy control mice. It was found that AST and ALT levels were significantly increased in ALF mice. The successful modelling establishment was indicated when focal necrosis and structural disorder in the hepatocytes of ALF mice were revealed by H&E staining, accompanied by hepatocellular oedema.

We then performed DEG analysis and enrichment analysis on the transcriptome sequencing data from the liver tissues of mice, and DEGs were shown to be significantly enriched in some immune-related pathways. The liver is a lymphoid organ with unique immune characteristics, and the balance between immune tolerance and immune activity is crucial to the physiological function of the liver. Therefore, the immune response has a great influence on the development of liver diseases (Chen & Tian, 2021). Many recent studies have shown that activation of systemic immune responses is central to the pathogenesis of ALF (Ye et al., 2022) Therefore, in this study, we compared the ratio of immune cells between groups from an immune perspective, and 4 types of immune cells were shown to be significantly different. The proportion of monocytes was significantly lower in the ALF group than that in the control group, while the proportions of macrophages M0, macrophages M1 and neutrophils were significantly higher in the ALF group than those in the control group. Monocytes was found to be involved in orchestrating the inflammatory response, thus intervening in ALF (Schneider et al., 2021), while a hepatic macrophage expressing MerTK was proved to be a potentially novel therapeutic target for ALF, because it could promote the regression of inflammation in ALF (Triantafyllou et al., 2018a). Dysfunction of monocytes as well as macrophages has been shown to be central to the progression of ALF (Triantafyllou et al., 2018b). Furthermore, the platelet receptor CLEC-2 was confirmed to block neutrophil-mediated liver recovery, contributing to the persistence and progression of liver injury in ALF (Chauhan et al., 2020). Our findings in the current study also further confirmed the important role of aforementioned immune cells in ALF. Overall, we finally obtained immune-related differential genes in ALF by performing WGCNA analysis on all the genes, clustering the gene modules significantly associated with the four immune cells, and taking intersections with the differential genes.

GO and KEGG enrichment analysis showed that these immune-related DEGs were revealed to be significantly enriched in some immune-related pathways, including TNF signalling pathway, NF-kappa B signalling pathway and NOD-like receptor signalling pathway. Tumour necrosis factor (TNF) is involved in the adaptive and innate immune system, and has been shown to be involved in immune system regulation by suppressing immune cell activity (Zhao et al., 2021). For example, the TNF- *α*/HMGB1 signalling pathway can be involved in pyroptosis in ALF (Wang et al., 2020), and targeting TNF- *α* not only attenuates the extent of hepatocyte injury in ALF but also enhances early regeneration of liver tissues (Lai et al., 2019). In addition, the NF-kappa B signalling pathway and the NOD-like receptor signalling pathway have also been shown to be involved in the development of ALF (Lv et al., 2018; Lyu et al., 2019). To further investigate the mechanisms of action of immune-related signalling pathways in depth, we selected the TNF signalling pathway for subsequent analysis. Genes enriched in the TNF signalling pathway were subjected to PPI analysis, and a total of three immune-related key genes (CXCL1, CXCL10 and IL1B) were finally obtained, and they were all revealed to be significantly upregulated in ALF.

CXCL1 and CXCL10 are members of the CXC subfamily of chemokines that can affect the disease progression by interfering with cytokine signalling in the immune system (Do, Lee & Cho, 2020). CXCL1 has chemotactic activity on neutrophils, and decreased CXCL1 levels can reduce the recruitment of neutrophils to liver tissue, thereby lessening the severity of liver injury (Kakizaki et al., 2021). CXCL10 can also bind to the receptor CXCR3 to stimulate monocyte migration to modulate the immune response, and has been shown to be a prognostic biomarker for immunotherapy of homologous recombination-deficient tumors (Shi et al., 2021). IL1B, a member of the interleukin-1 cytokine family, is derived from activated macrophages and is typically involved in a variety of cellular activities that mediate inflammatory responses (El-Sharkawy, Brough & Freeman, 2020). Besides, IL1B has also been recognized as a marker of liver inflammation for the determination of the severity of liver injury (Fan et al., 2021). However, it remains unclear what is the mechanism of action of these three genes influencing ALF through mediating immunity. The present study investigated and finally revealed that CXCL1, CXCL10 and IL1B could mediate immunity and thus influence the disease progression in ALF by participating in the TNF signalling pathway. At the same time, this study suggested that the above three key genes may not only serve as biomarkers of inflammatory response but also as potential key targets for the immunotherapy of ALF. In general, the findings of the current research may provide a possible therapy target for the treatment of ALF, and may also construct a reliable scientific basis for the immunotherapy of ALF.

However, this study also has shortcomings and limitations. Firstly, key immune-related genes were only investigated in the ALF mouse model, but not further validated at the clinical level, so their clinical significance remains to be confirmed. Secondly, only the TNF signalling pathway was selected for analysis, and the results may be relatively limited. Thirdly, this study did not explore the mechanism of the key genes involved in the signalling pathway that mediates immunity.

## Conclusion

Key immune-related genes in ALF were identified in this study, which may provide not only potential therapeutic targets for treating ALF and improving its prognosis, but also a reliable scientific basis for the immunotherapy of the disease.

##  Supplemental Information

10.7717/peerj.15241/supp-1 Table S1 Top 10 genes in PPI network ranked by 5 algorithms

10.7717/peerj.15241/supp-2 Data S1 Raw data for Figures 1, 6, 10

10.7717/peerj.15241/supp-3 Supplemental Information 3 ARRIVE 2.0 checklist

10.7717/peerj.15241/supp-4 Supplemental Information 4 Sequence data

## Additional Information and Declarations

### DNA Deposition

The following information was supplied regarding the deposition of DNA sequences: All the sequence data is available at GEO: GSE217659.

### Data Availability

The following information was supplied regarding data availability: The raw data are available in the Supplemental Files.
