# Supplementary figures and images for: Identification of key immune-related genes associated with LPS/D-GalN-induced acute liver failure in mice based on transcriptome sequencing
Source: PeerJ. 2023 May 5;11:e15241. doi: 10.7717/peerj.15241 (PMC10166078; doi:10.7717/peerj.15241)

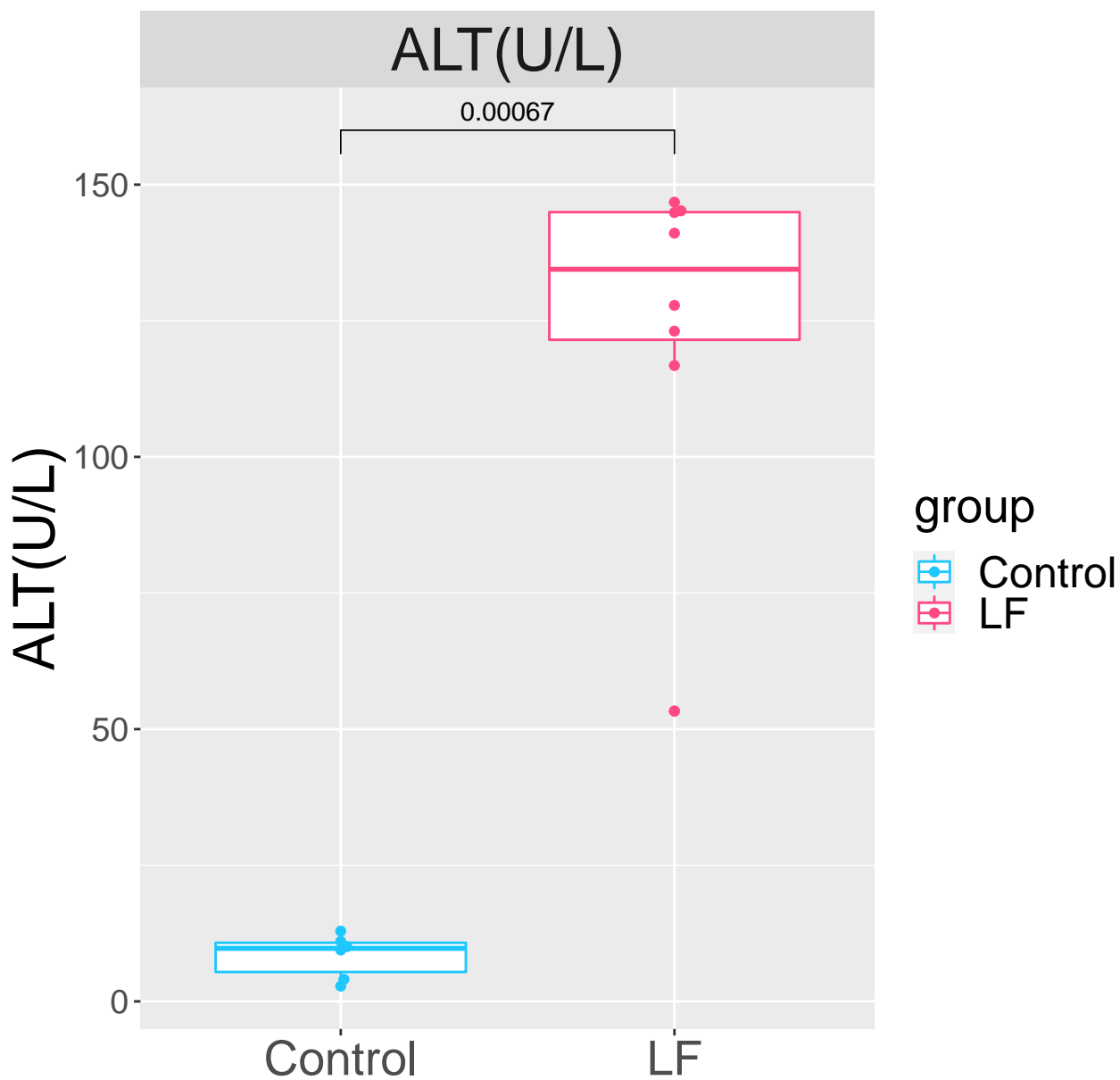

Supplement: Data S1 [file peerj-11-15241-s002.zip › Raw Data/Figure 1/ALT_diff.pdf]

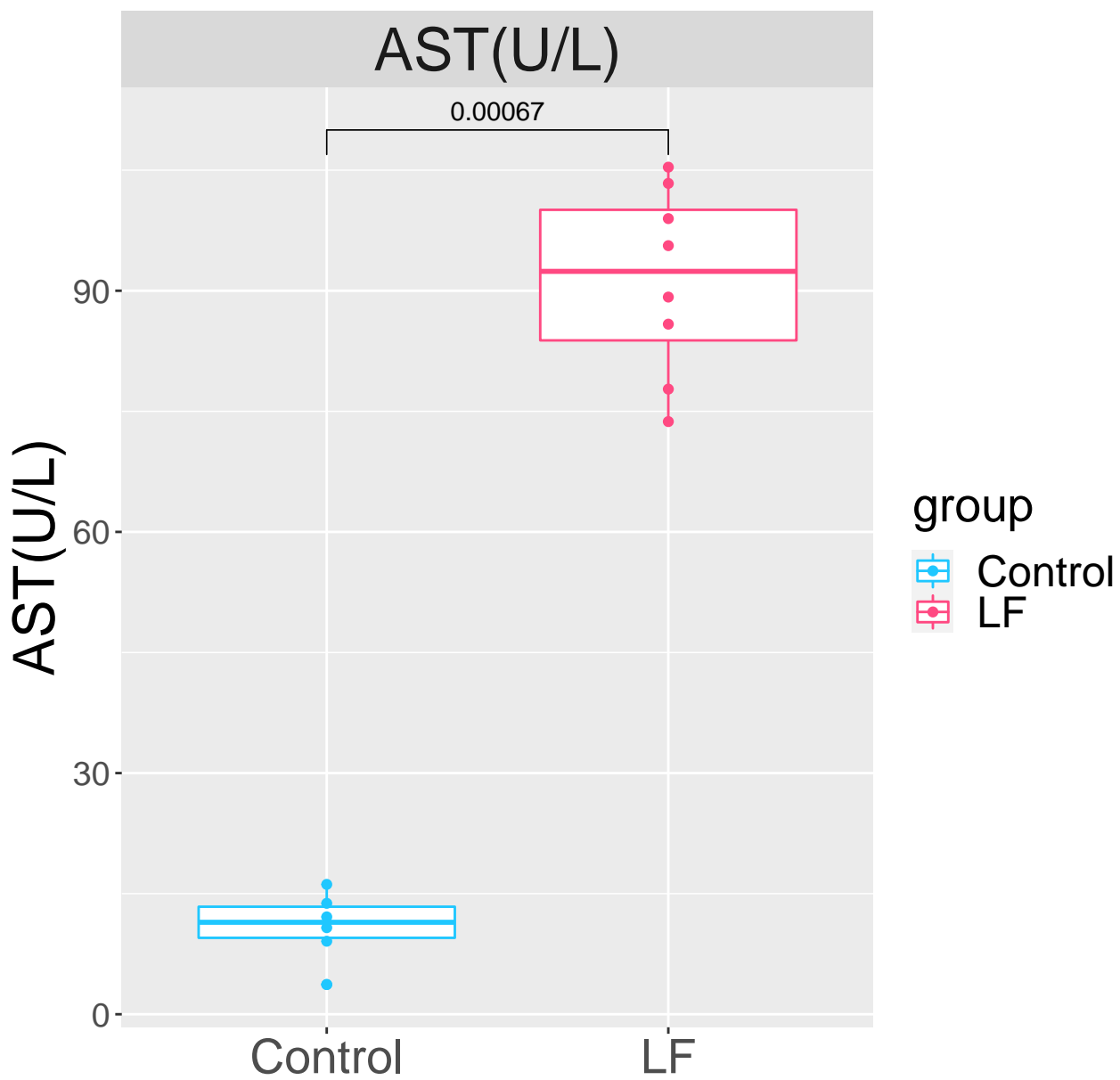

Supplement: Data S1 [file peerj-11-15241-s002.zip › Raw Data/Figure 1/AST_diff.pdf]

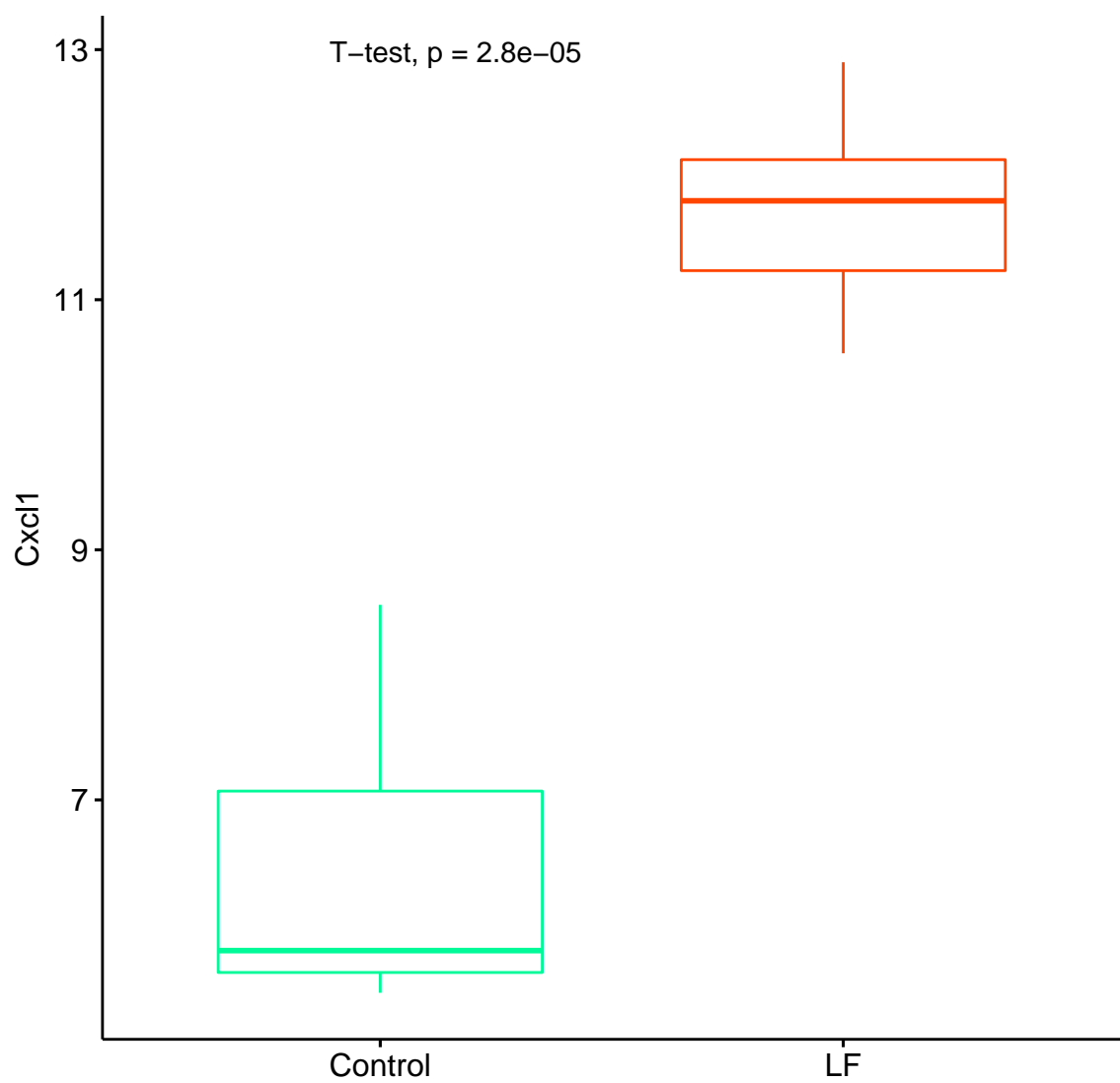

Supplement: Data S1 [file peerj-11-15241-s002.zip › Raw Data/Figure 10/boxplot-diffexp.Cxcl1.pdf]

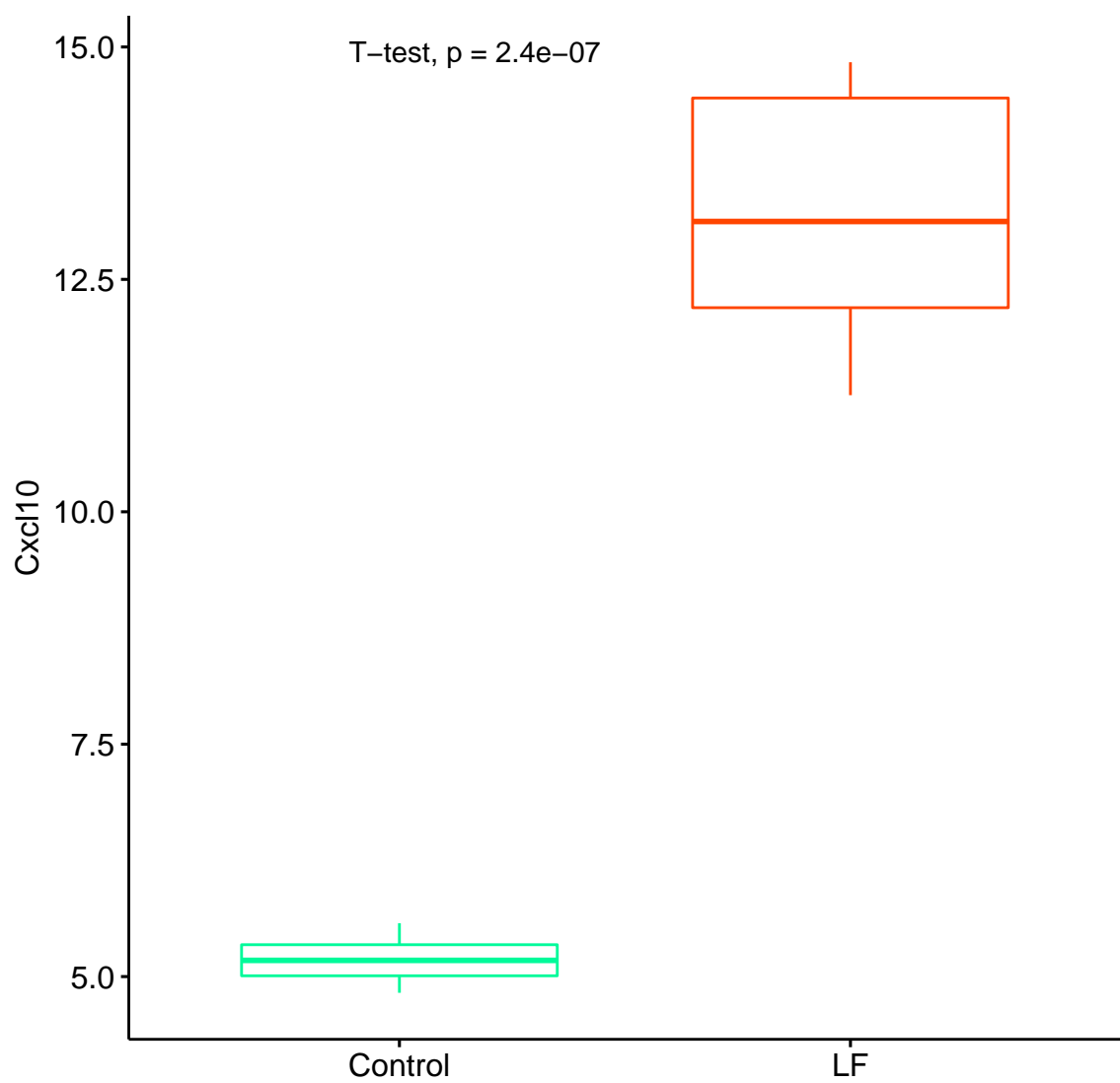

Supplement: Data S1 [file peerj-11-15241-s002.zip › Raw Data/Figure 10/boxplot-diffexp.Cxcl10.pdf]

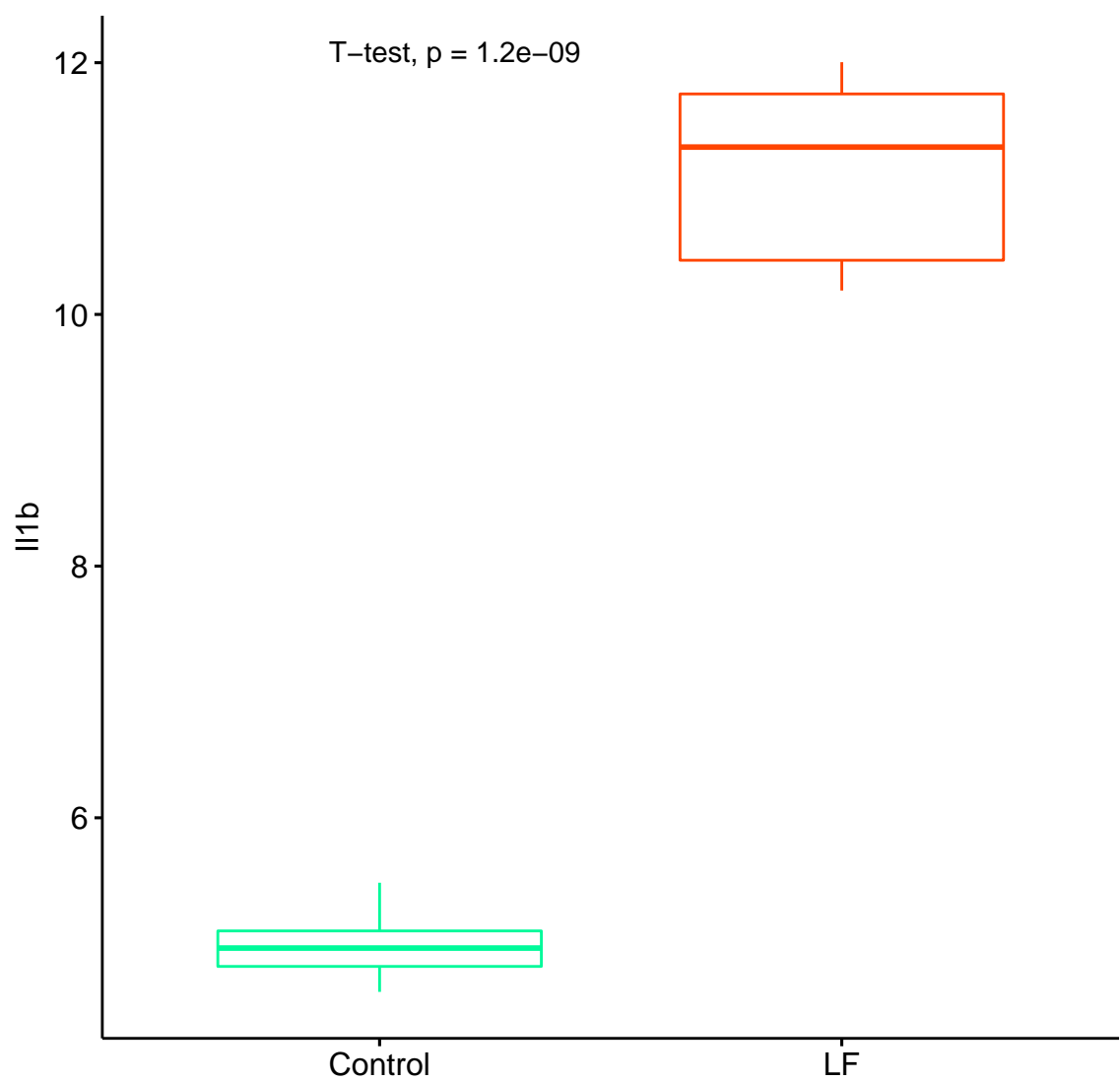

Supplement: Data S1 [file peerj-11-15241-s002.zip › Raw Data/Figure 10/boxplot-diffexp.Il1b.pdf]

# Macrophages.M0

Cell fraction

0.20

0.15

0.10

0.05

0.0013

Control

LF

group

Control  
LF

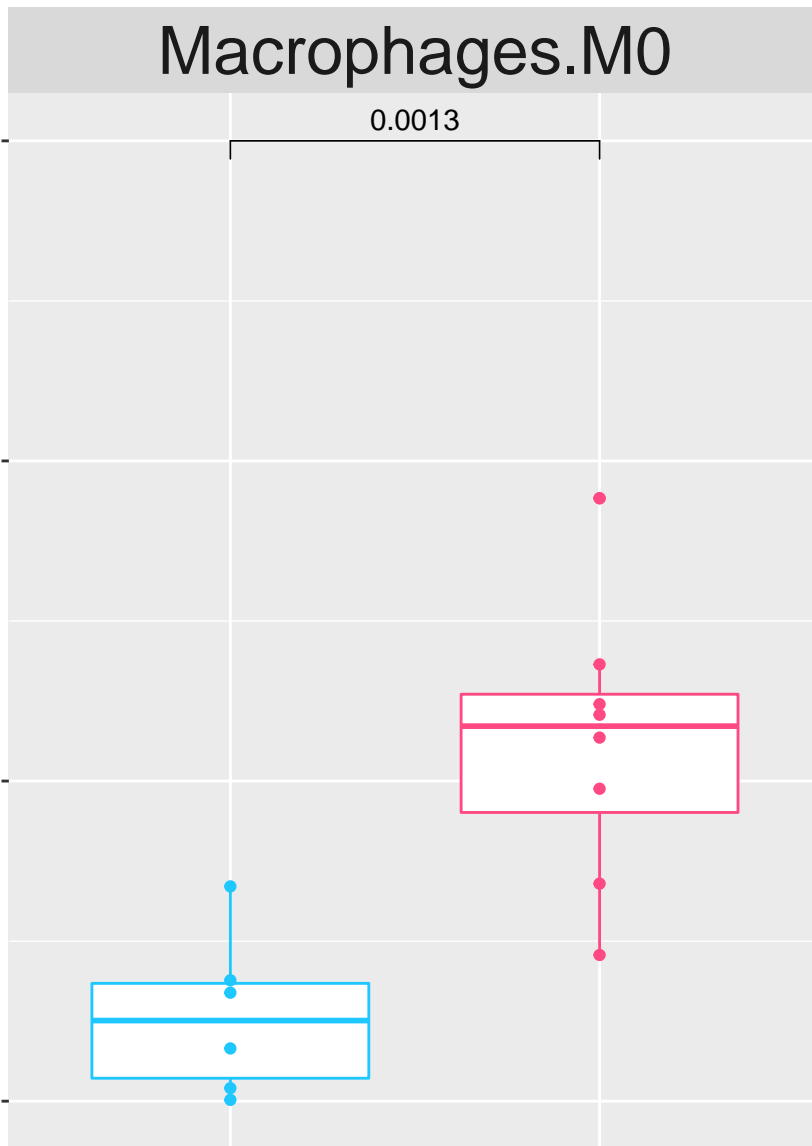

Supplement: Data S1 [file peerj-11-15241-s002.zip › Raw Data/Figure 6/Macrophages.M0_diff.pdf]

# Macrophages.M1

Cell fraction

0.00067

0.4

0.3

0.2

0.1

0.0

Control

LF

group

Control  
LF

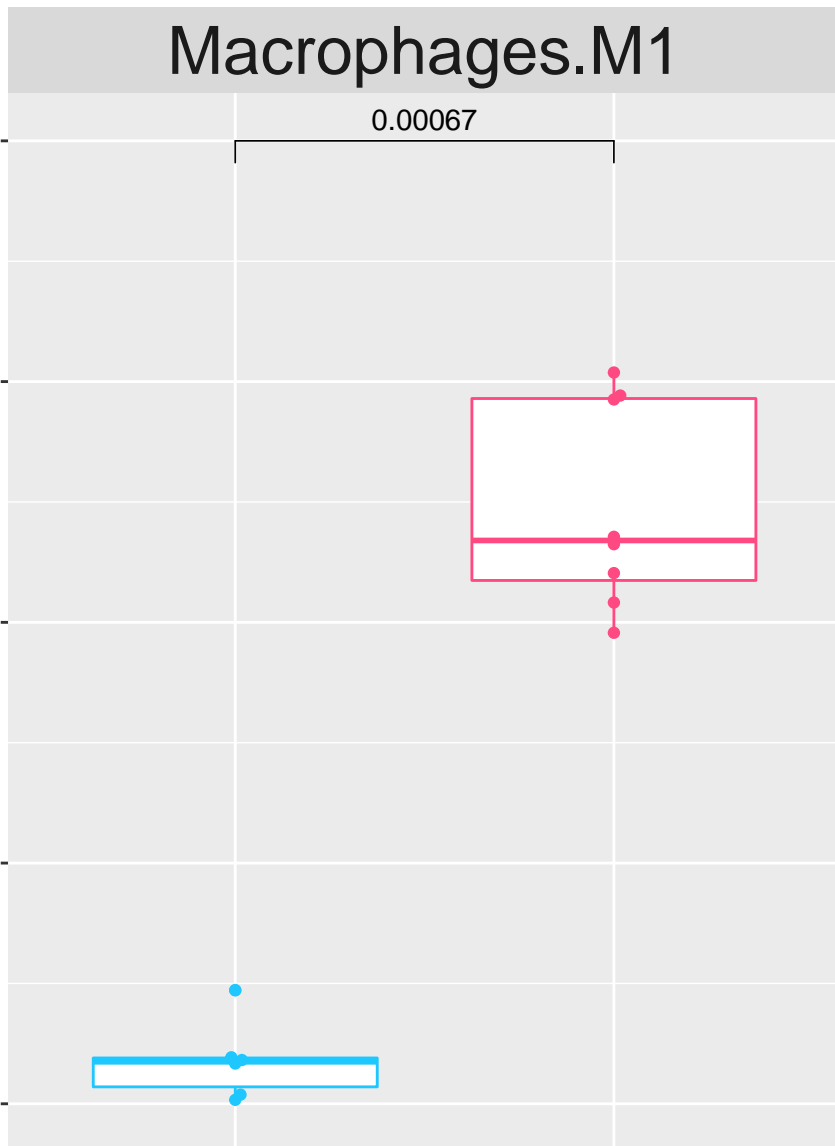

Supplement: Data S1 [file peerj-11-15241-s002.zip › Raw Data/Figure 6/Macrophages.M1_diff.pdf]

# Monocytes

Cell fraction

0.00067

0.6

0.4

0.2

Control

LF

group

Control  
LF

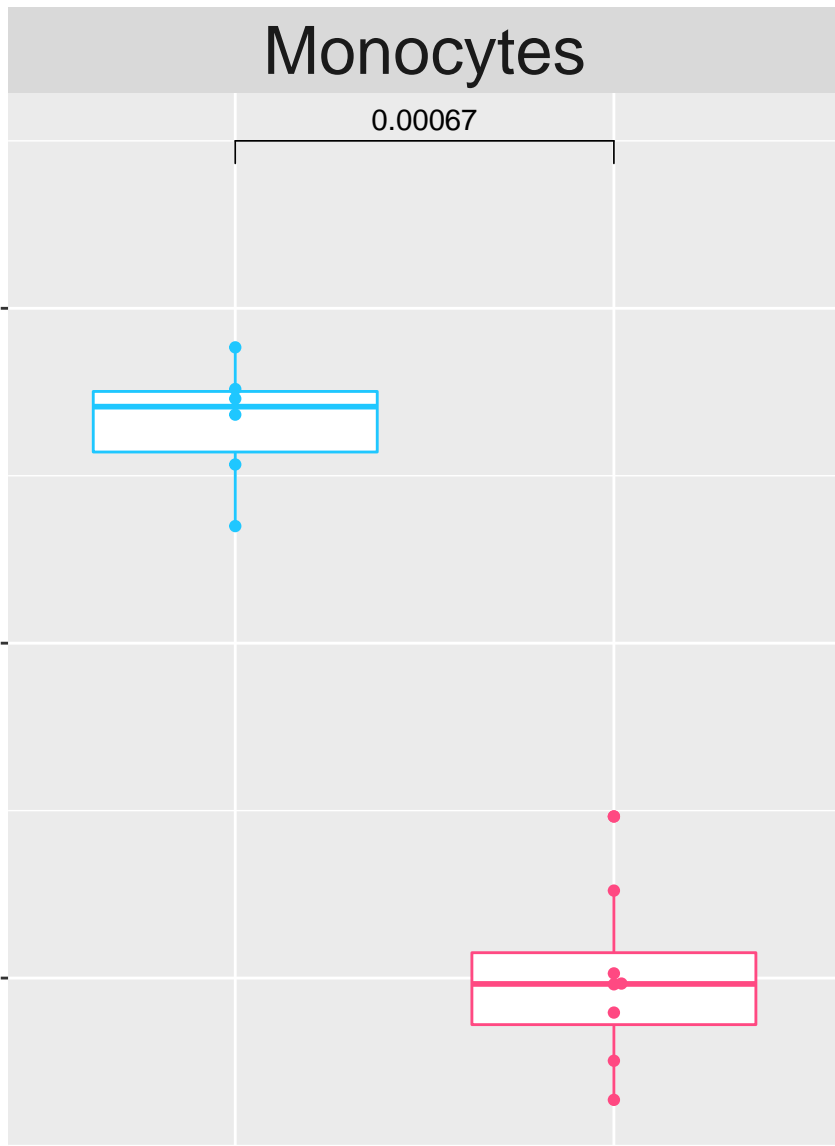

Supplement: Data S1 [file peerj-11-15241-s002.zip › Raw Data/Figure 6/Monocytes_diff.pdf]

# Neutrophils

Cell fraction

0.4  
0.3  
0.2  
0.1  
0.0

0.0016

Control

LF

group

Control  
LF

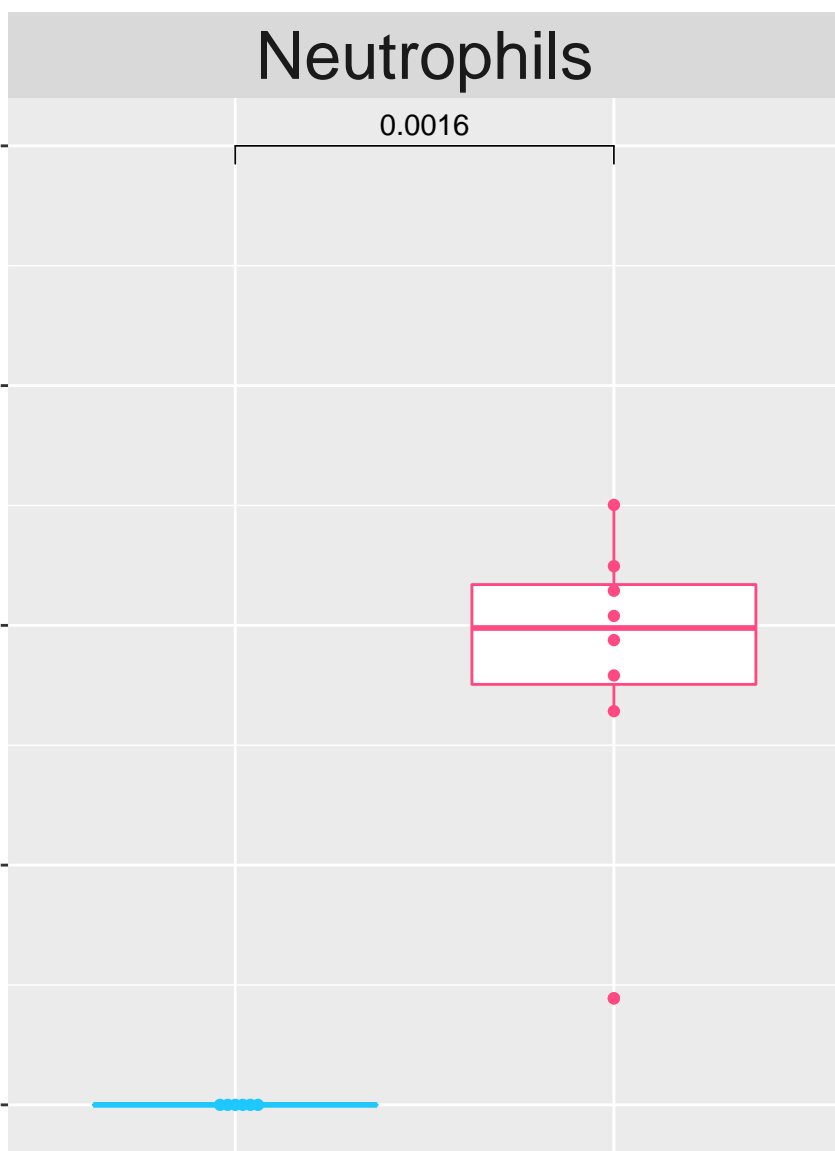

Supplement: Data S1 [file peerj-11-15241-s002.zip › Raw Data/Figure 6/Neutrophils_diff.pdf]
