# Supplementary material for: Identification of key immune-related genes associated with LPS/D-GalN-induced acute liver failure in mice based on transcriptome sequencing
Source: PeerJ. 2023 May 5;11:e15241. doi: 10.7717/peerj.15241 (PMC10166078; doi:10.7717/peerj.15241)
Supplement: Supplemental Information 4 [file peerj-11-15241-s004.doc]

Dear editors and reviewers,

All the sequence data has been deposited in the GEO database.

You may view the deposited data at:

<https://www.ncbi.nlm.nih.gov/geo/query/acc.cgi?acc=GSE217659>

accession number: GSE217659

access token: ulmvsqsylzkllqj

If there are any problems, please feel free to contact me.

Yours sincerely,

Hong Wang
