# Supplementary material for: Identification of key immune-related genes associated with LPS/D-GalN-induced acute liver failure in mice based on transcriptome sequencing
Source: PeerJ. 2023 May 5;11:e15241. doi: 10.7717/peerj.15241 (PMC10166078; doi:10.7717/peerj.15241)
Supplement: Data S1 [file peerj-11-15241-s002.zip › Raw Data/Figure 1/ALT&AST.doc]

**ALT&AST**

Date：2021.12.2.

Animal：C57BL/6 male mice

| **control group** | **ALF group** |  |
| --- | --- | --- |
| 2021.12.2-C57  mouse-liver failure model-control group-1 23.5g | M1 23g | M7 22g |
| 2021.12.2-C57  mouse-liver failure model-control group-2 24.5g | M2 23g | M8 23g |
| 2021.12.2-C57  mouse-liver failure model-control group-3 25g | M3 23g |  |
| 2021.12.2-C57  mouse-liver failure model-control group-4 24g | M4 21.5g |  |
| 2021.12.2-C57  mouse-liver failure model-control group-5 25g | M5 25g |  |
| 2021.12.2-C57  mouse-liver failure model-control group-6 25g | M6 24g |  |

**Serum for the detection of ALT and AST**

| AST standard curve | | | | | |
| --- | --- | --- | --- | --- | --- |
| OD value | 0.28 | 0.356 | 0.419 | 0.476 | 0.556 |
| absolute OD value | 0 | 0.076 | 0.139 | 0.196 | 0.276 |
| enzyme activity (karmen unit) | 0 | 24 | 61 | 114 | 190 |

| AST | | | | |
| --- | --- | --- | --- | --- |
| blank group-OD value | control group-OD value | absolute OD value | enzyme activity (karmen unit) | U/L |
| 0.316 | 0.369 | 0.053 | 18.84697 | 9.08423954 |
| 0.316 | 0.353 | 0.037 | 7.67113 | 3.69748466 |
| 0.316 | 0.378 | 0.062 | 25.13338 | 12.11428916 |
| 0.316 | 0.374 | 0.058 | 22.33942 | 10.76760044 |
| 0.316 | 0.39 | 0.074 | 33.51526 | 16.15435532 |
| 0.316 | 0.383 | 0.067 | 28.62583 | 13.79765006 |
|  |  |  |  |  |
| blank group-OD value | ALF group-OD value | absolute OD value | enzyme activity (karmen unit) | U/L |
| 0.316 | 0.607 | 0.291 | 185.08759 | 89.21221838 |
| 0.316 | 0.561 | 0.245 | 152.95705 | 73.7252981 |
| 0.316 | 0.655 | 0.339 | 218.61511 | 105.372483 |
| 0.316 | 0.649 | 0.333 | 214.42417 | 103.3524499 |
| 0.316 | 0.573 | 0.257 | 161.33893 | 77.76536426 |
| 0.316 | 0.636 | 0.32 | 205.3438 | 98.9757116 |
| 0.316 | 0.597 | 0.281 | 178.10269 | 85.84549658 |
| 0.316 | 0.626 | 0.31 | 198.3589 | 95.6089898 |

| ALT standard curve | | | | | | |
| --- | --- | --- | --- | --- | --- | --- |
| OD value | 0.279 | 0.331 | 0.387 | 0.473 | 0.523 | 0.572 |
| absolute OD value | 0 | 0.052 | 0.108 | 0.194 | 0.244 | 0.293 |
| enzyme activity (karmen unit) | 0 | 28 | 57 | 97 | 150 | 200 |

| ALT | | | | |
| --- | --- | --- | --- | --- |
| blank group-OD value | control group-OD value | absolute OD value | enzyme activity (karmen unit) | U/L |
| 0.29 | 0.335 | 0.045 | 20.86835 | 10.0585447 |
| 0.29 | 0.312 | 0.022 | 5.8022 | 2.7966604 |
| 0.29 | 0.338 | 0.048 | 22.8335 | 11.005747 |
| 0.29 | 0.344 | 0.054 | 26.7638 | 12.9001516 |
| 0.29 | 0.333 | 0.043 | 19.55825 | 9.4270765 |
| 0.29 | 0.316 | 0.026 | 8.4224 | 4.0595968 |
|  |  |  |  |  |
| blank group-OD value | ALF group-OD value | absolute OD value | enzyme activity (karmen unit) | U/L |
| 0.29 | 0.472 | 0.182 | 110.6102 | 53.3141164 |
| 0.29 | 0.763 | 0.473 | 301.22975 | 145.1927395 |
| 0.29 | 0.673 | 0.383 | 242.27525 | 116.7766705 |
| 0.29 | 0.693 | 0.403 | 255.37625 | 123.0913525 |
| 0.29 | 0.708 | 0.418 | 265.202 | 127.827364 |
| 0.29 | 0.768 | 0.478 | 304.505 | 146.77141 |
| 0.29 | 0.75 | 0.46 | 292.7141 | 141.0881962 |
| 0.29 | 0.762 | 0.472 | 300.5747 | 144.8770054 |
